# Promoted Current Injection in GaInN/GaN Multi‐Quantum Nanowire‐Based LEDs Coated With AZO Using Atomic Layer Deposition

**DOI:** 10.1002/nap2.70195

**Published:** 2026-07-07

**Authors:** Soma Inaba, Mengtong Wang, Weifang Lu, Kazuma Ito, Sena Miura, Yiyu Ou, Dong‐Pyo Han, Kai Huang, Motoaki Iwaya, Tetsuya Takeuchi, Rong Zhang, Satoshi Kamiyama

**Affiliations:** ^1^ Department of Materials Science and Engineering Meijo University Nagoya Japan; ^2^ Fujian Key Laboratory of Semiconductor Materials and Applications, CI Center for OSED, Department of Physics Xiamen University Xiamen China; ^3^ Future Display Institute in Xiamen Xiamen China; ^4^ Department of Photonics Engineering Technical University of Denmark Kgs. Lyngby Denmark; ^5^ Display & Semiconductor Engineering, School of Electrical Engineering Pukyong National University (PKNU) Busan Republic of Korea

**Keywords:** atomic layer deposition, AZO, current injection, finite element simulation, GaInN/GaN, NW‐LED

## Abstract

GaInN/GaN multi‐quantum shell (MQS) nanowires (NWs) are promising candidates for high‐efficiency microscale and nanoscale light‐emitting diodes (LEDs), offering reduced efficiency losses from the quantum‐confined Stark effect and nonradiative recombination. However, the three‐dimensional NW geometry complicates electrode deposition, leading to nonuniform current injection and emission variations. Here, we demonstrated enhanced current injection in NW‐LEDs using a conformal aluminum‐doped zinc oxide (AZO) film deposited via atomic layer deposition (ALD). Finite element simulations revealed that extended p‐type electrode coverage can promote both axial and lateral current injection from the sidewalls, enhancing radiative recombination across the (0001), (1$\bar{1}$01), and (10$\bar{1}$0) planes. We systematically compared device fabrication processes, transparent electrode morphologies, and optoelectronic performance using AZO and indium tin oxide (ITO) electrodes deposited via ALD and sputtering. Cross‐sectional morphology characterization confirmed minimal thickness variation of ALD‐deposited AZO, enabling uniform sidewall coverage and deposition in the regions between NWs, which is challenging for sputtered ITO. Uniform AZO deposition by ALD improved light output by a factor of 1.2 at an injection current of 30 mA. These results demonstrate that conformal AZO enables uniform transparent electrodes, promotes efficient current injection throughout the NWs, and represents a promising alternative to ITO in NW‐LEDs.

## Graphical abstract

Conformal Al‐doped ZnO (AZO) electrodes deposited by atomic layer deposition (ALD) overcome current‐injection limitations in coaxial GaInN/GaN nanowire LEDs. Uniform electrode coverage across nanowire sidewalls and bottom regions promotes efficient carrier injection and radiative recombination across multiple crystal facets, enhancing emission uniformity and light output. The results establish ALD‐AZO as a promising transparent electrode for high‐performance nanowire LEDs.

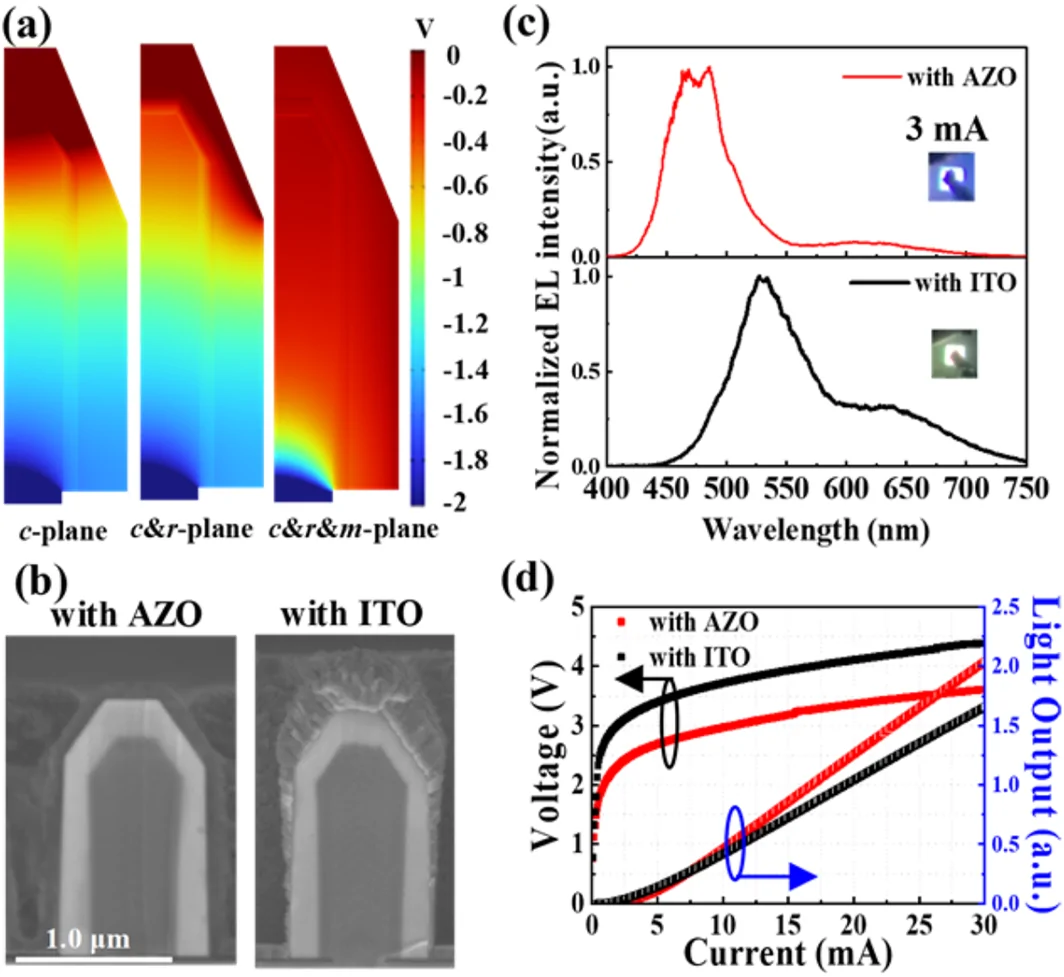

## 1 Introduction

With the development of display technologies, organic‐based light‐emitting diode (OLED) and inorganic semiconductor‐based microscale LED (micro‐LED) displays are being explored as viable alternatives to the traditional liquid crystal displays (LCD). Group III‐nitride‐based micro‐LEDs have garnered worldwide attention for their revolutionary impact on visual display systems, augmented reality (AR), virtual reality (VR), and other technologies, as they offer promising potential for enhancing energy efficiency, achieving vibrant color reproduction, enhancing contrast ratios, and extending lifetime [1, 2, 3, 4, 5, 6, 7]. However, there still remain several challenges that need to be resolved. The primary issues include the quantum‐confined Stark effect (QCSE) and a decrease in luminous efficiency due to high Shockley–Read–Hall (SRH) nonradiative recombination rates resulting from defects and damage to the mesa sidewalls induced during dry etching [8, 9, 10, 11]. To overcome these limitations and realize high‐performance micro‐LEDs, increasing attention has been devoted to nanowire (NW)‐based LEDs grown via bottom‐up approaches, including both dot‐in‐NW structures [12, 13, 14] and GaInN/GaN core–shell NWs [15, 16, 17, 18]. Recent studies have demonstrated the excellent performance of dot‐in‐NW LEDs for microdisplay applications [12, 13, 14]. In contrast, GaInN/GaN NWs exhibit a three‐dimensional growth configuration, forming hexagonal prismatic microcrystals with a structure consisting of an n‐GaN core, GaInN/GaN multi‐quantum shells (MQSs), and a p‐GaN shell [19, 20, 21]. The structural properties of NW‐LEDs enable the utilization of nonpolar ($10\bar{1}0$) and semipolar ($1\bar{1}01$) sidewalls of the NWs as the active layer, effectively reducing the QCSE [22]. In the fabrication process of NW‐LEDs, owing to the independent structure of each NW, the device structure can be designed to prevent current injection into the NWs damaged by dry etching at the outermost periphery, whereas the exposure of mesa sidewalls through dry etching can also be minimized. These characteristics make core–shell NWs a promising platform for high‐efficiency micro‐LEDs [20]. Furthermore, the commercial potential of this technology has been demonstrated by recent developments from Aledia, which has reported significant progress toward large‐scale manufacturing of core–shell NW micro‐LEDs for display applications [23, 24].

Nevertheless, the three‐dimensional structure of NWs presents a challenge in achieving uniform transparent electrode deposition during LED fabrication [25]. For instance, when a transparent indium tin oxide (ITO) electrode is deposited via sputtering, the ITO tends to accumulate near the NW tips and upper sidewalls, whereas only a very thin coating is formed on the lower sidewalls and in the regions between neighboring NWs. This nonuniform electrode coverage leads to uneven current injection and carrier distribution within the NWs, resulting in different dominant emission regions and emission wavelengths even under the same injection current density. Furthermore, the ultrathin ITO layer near the NW base limits lateral current spreading, contributes to localized dot‐like luminescence, and increases the operating voltage. These limitations primarily originate from the shadowing effect inherent to the line‐of‐sight sputtering process. Although optimization of the sputtering conditions can partially improve electrode coverage, as demonstrated in our previous work [25], achieving highly conformal transparent electrodes on three‐dimensional NW structures remains a significant challenge. Atomic layer deposition (ALD) offers a promising alternative for achieving conformal transparent electrodes on nanostructures due to its cyclic, ångström‐by‐ångström growth mechanism [26, 27]. Unlike conventional chemical vapor deposition, where films form through direct gas‐phase decomposition, ALD enables precise, self‐limiting growth of atomic layers. For instance, ALD‐grown aluminum‐doped zinc oxide (AZO) thin films exhibit a work function range of 4.48–4.65 eV and a high transmittance exceeding 90% in the visible light range (380–900 nm) [28, 29, 30, 31]. These properties make AZO a promising alternative to traditional ITO in optoelectronic applications, particularly for nanostructure‐based technologies such as flexible electronics and photovoltaics [32, 33, 34]. Moreover, considering the scarcity of indium (In), the abundance and cost‐effectiveness of the materials used in AZO fabrication offer a sustainable alternative. However, reports on AZO‐based LEDs remain limited, and the performance of AZO in NW‐LEDs has yet to be thoroughly explored. Furthermore, the effects of nonuniform current injection caused by uneven ITO coverage on the NW sidewalls have not been thoroughly analyzed, leaving the underlying physical mechanisms unclear. Therefore, a detailed investigation of the spatially resolved optical and electrical behaviors of core–shell NW‐LEDs is crucial for addressing these nonuniformity issues and improving device performance.

In this study, sputtered ITO and ALD‐grown AZO layers were systematically investigated as transparent electrodes for NW‐based LEDs to elucidate their respective effects on device performance. Finite element simulations were employed to visualize and quantify carrier distribution, electric field modulation, and current pathways within NW structures under different electrode geometries. These simulations not only provide an intuitive understanding of carrier injection mechanisms but also serve as a guide for optimizing structural design and electrode engineering in NW‐LEDs. Particular attention was given to the intrinsic differences between sputtering and ALD processes on NW structures. The morphological characteristics of the fabricated samples were examined using scanning electron microscopy (SEM), whereas the optical and electrical properties were evaluated through electroluminescence (EL) spectra and current–voltage–light output (I–V–L) measurements. By correlating the structural features observed with the optical and electrical properties, a comprehensive understanding of the influence of transparent electrode materials on NW‐LED performance was achieved. The consistency between the experimental and theoretical calculation results validates this approach and provides a solid foundation for the optimization of electrode designs in NW‐based optoelectronic devices.

## 2 Experimental and Simulation Section

GaN/sapphire templates featuring triangular patterns, with a diameter of 320 nm and a pitch of 1200 nm, were fabricated on a SiO_2_ mask by utilizing nanoimprint lithography and an inductively coupled plasma (ICP) etching process. The growth processes were performed in metal‐organic chemical vapor deposition (MOCVD) equipment (SR 4000, TAIYO NIPPON SANSO Co., Shinagawa‐ku, Tokyo, Japan) with the sequential formation of n‐GaN core, GaInN/GaN superlattices (SLs), GaInN/GaN MQS, and p‐GaN shell [35, 36, 37]. Table 1 shows the detailed growth conditions of Samples p and a–f. To confirm the uniformity of ALD deposition, the growth time of the n‐GaN core for Samples a and b was 95 s, accompanied by the introduction of 20 pairs of SLs, resulting in tall NWs with a large sidewall area. For comparison, Sample p was grown under the same conditions as Sample b but without SL insertion. In Samples c–f, the n‐GaN core growth time was shortened to 70 s, whereas the number of SL pairs was increased to 40. The growth conditions for the five pairs of GaInN/GaN MQSs, including AlGaN spacers in Samples a and b, included GaInN wells at 750°C for 4.3 min, GaN barriers at 810°C for 8.0 min, and AlGaN spacers at 750°C for 50 s, all under a pressure of 90 kPa [38]. For Samples c–f, the growth time of the GaN barriers was extended from 8 to 24 min [36]. Identical growth conditions were applied to the p‐GaN shell in all samples, with a low V/III ratio of 557 for 3 min followed by a high V/III ratio of 1115 for 30 s. AZO films deposited by ALD were employed as transparent electrodes for Samples a, c, and e, whereas ITO films deposited by sputtering were used for Samples p, b, d, and f for comparison.

**TABLE 1:** NW samples with different growth conditions.

Sample	Temperature and growth time of the core	Number of SL pairs	Temperature and growth time of GaInN in MQS	Temperature and growth time of GaN in MQS	AZO or ITO	Thermal annealing	Device size (µm^2^)
p	1150°C, 95 s	0	750°C, 4.3 min	810°C, 8.0 min	ITO	600°C, 4 min	340 × 340
a	1150°C, 95 s	20	750°C, 4.3 min	810°C, 8.0 min	AZO	Without	50 × 50
b	1150°C, 95 s	20	750°C, 4.3 min	810°C, 8.0 min	ITO	600°C, 4 min	50 × 50
c	1150°C, 70 s	40	750°C, 4.3 min	810°C, 24.0 min	AZO	Without	100 × 100
d	1150°C, 70 s	40	750°C, 4.3 min	810°C, 24.0 min	ITO	600°C, 4 min	100 × 100
e	1150°C, 70 s	40	750°C, 4.3 min	810°C, 24.0 min	AZO	650°C, 4 min	50 × 50
f	1150°C, 70 s	40	750°C, 4.3 min	810°C, 24.0 min	ITO	650°C, 4 min	50 × 50

In Sample a, the ALD‐AZO region was patterned by a photoresist mask, whereas in Samples c and e, patterning was achieved by dry etching. Therefore, the device structures and fabrication processes of Samples a, b, and c–f were different, as shown in Figure 1. Figure 1a–e schematically shows the device fabrication process for Sample a. After NW growth, the Mg acceptors in the p‐GaN shell were activated within the MOCVD chamber at 650°C for 30 min under a nitrogen (N_2_) atmosphere. A Ni mask was then applied to the designated mesa region using electron beam deposition (EB, MA08‐3065, ULVAC, Chigasaki, Kanagawa, Japan), followed by selective removal of NWs in other areas by ICP etching. To restrict AZO deposition to the mesa area, a resist mask was applied prior to deposition at 200°C in an ALD reactor (Model R200, PICOSUN, Finland). The ALD conditions, summarized in Table 2, involved alternating cycles of diethylzinc (DEZ) → H_2_O and trimethylaluminum (TMA) → H_2_O. A total of 44 macrocycles were applied to form a 150‐nm‐thick AZO layer, with each macrocycle consisting of 19 DEZ → H_2_O cycles and one TMA → H_2_O cycle. The AZO‐coated photoresist mask was subsequently removed by immersing in a developer solution heated to 70°C for 24 h, followed by ultrasonic cleaning for 5 min. Finally, Cr/Ni/Au metal contacts (10/20/200 nm) were sequentially deposited on the n‐ and p‐electrodes using electron beam evaporation. Sample b was fabricated using the same process flow as Sample a, with the only difference being that the transparent electrode was deposited by ITO sputtering. In this case, a 150‐nm‐thick ITO layer was deposited using high‐frequency magnetron sputtering (CFS‐4EP, Shibaura Mechatronics Co., Japan), followed by rapid thermal annealing at 600°C for 4 min under a N_2_ atmosphere (RTA, MR094017‐0, ULVAC Riken, Tsuzuki‐ku, Tokyo). Figure 1f–j schematically illustrates the fabrication process of Sample c, in which AZO deposition was performed after mesa patterning by dry etching. The activation annealing, mesa etching, and Cr/Ni/Au electrode deposition steps were identical to those used for Sample a. To further validate the NW‐LED fabrication process and minimize the influence of boundary NWs, a SiO_2_ isolation layer was introduced along the mesa edge prior to AZO deposition. The SiO_2_ layer prevented direct contact between AZO and the exposed n‐GaN surface, thereby electrically isolating the peripheral regions and suppressing unintended current injection. Subsequently, AZO was deposited over the entire sample by ALD under the identical conditions as those for Sample a, followed by selectively removing the AZO outside the mesa regions. Sample d was fabricated following the same process flow as Sample c, and Sample f followed the same process flow as Sample e, with sputtered ITO. The mesa sizes were 340 × 340 μm^2^ for Sample p, 100 × 100 μm^2^ for Samples c and d, and 50 × 50 μm^2^ for the other samples. Based on the experimental results of Samples a–d, the contact resistance between AZO and p‐GaN annealed by RTA was investigated in a planar LED using a transmission line model (TLM) test structure. The AZO annealing was conducted at temperatures of 600°C and 650°C under a N_2_ atmosphere. Based on the above results, after AZO deposition, NW Sample e was also annealed at 650°C for 4 min, whereas the other processes remained the same as those for Sample c. The device structure of NW‐LEDs with AZO and ITO was evaluated by SEM, and the emission characteristics of the NWs were assessed through EL and I–V–L measurements.

**FIGURE 1:**
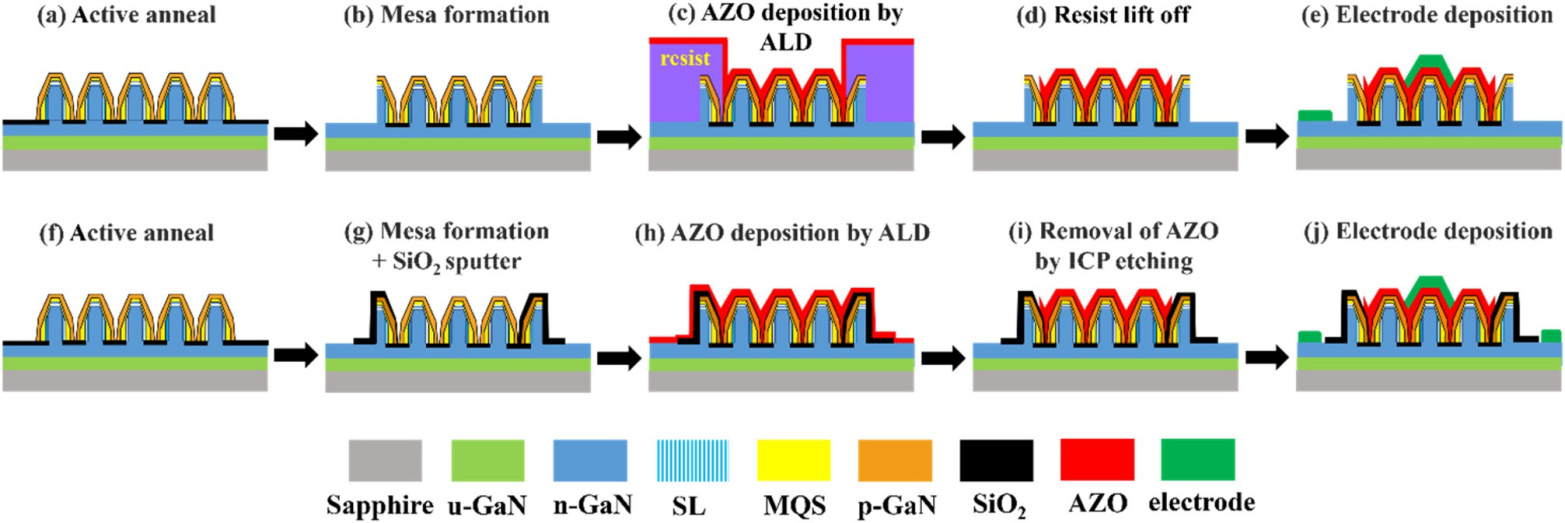
Schematic diagrams of fabrication processes for Sample a: (a) activation annealing in a N_2_ atmosphere; (b) Ni deposition in the mesa region and removal of NWs outside the mesa by ICP‐based Cl_2_ etching; (c) resist coating and AZO deposition by ALD; (d) resist lift‐off by developer solution for 24 h; (e) electrode deposition. Schematic diagrams of Sample c: (f) activation annealing; (g) mesa formation and SiO_2_ sputtering at the edge of the mesa; (h) AZO deposition by ALD on the entire surface of the sample; (i) removal of AZO outside the mesa by ICP‐based BCl_3_ etching; (j) electrode deposition.

**TABLE 2:** AZO deposition conditions by ALD.

Precursor	Carrier gas (N_2_) flow (sccm)	Pulse time (sec)	Purge time (sec)	Cycles
DEZ	150	0.1	5	19
H_2_O	200	0.1	5	
TMA	150	0.1	5	1
H_2_O	200	0.1	5	

To investigate the effect of p‐type contact position on the optoelectronic performance of NW‐LEDs, three device models were designed with p‐type contacts located, respectively, on the (0001) plane, on the (0001) and $\left(1\bar{1}01\right)$ planes, and on the (0001), $\left(1\bar{1}01\right)$, and $\left(10\bar{1}0\right)$ planes, as illustrated in Table 3. To isolate the effect of contact geometry, a uniform In content was adopted in the GaInN quantum wells. The NW structure, from top to bottom, consisted of a 125‐nm‐thick p‐GaN shell doped with Mg at 5 × 10^18^ cm^−3^, MQSs, and an n‐GaN core of 300 nm in thickness and 800 nm in height with Si doping of 5 × 10^18^ cm^−3^. The MQS structure consisted of five GaN (5 nm)/Ga_0.8_In_0.2_N (3 nm) pairs. Finite element simulations based on density gradient theory were employed to accurately model carrier transport and recombination in the NW‐LED structures. An Auger recombination coefficient of 1.7 × 10^−31^ cm^6^ s^−1^ was used to analyze Auger processes [39, 40], whereas SRH lifetimes of electrons and holes were set to 100 ns [41]. Polarization was set to 40% to approximate interface charge effects [42, 43], considering partial relaxation due to dislocations generated during growth. The electron and hole mobilities were set to 300 and 10 cm^2^/V/s, respectively [44].

**TABLE 3:** The parameters of the samples used for simulation.

Sample	(0001) plane	$\left(1\bar{1}01\right)$ plane	$\left(10\bar{1}0\right)$ plane	p‐contact position
A	Ga_0.8_In_0.2_N	Ga_0.8_In_0.2_N	Ga_0.8_In_0.2_N	(0001) plane
B	Ga_0.8_In_0.2_N	Ga_0.8_In_0.2_N	Ga_0.8_In_0.2_N	(0001) and $\left(1\bar{1}01\right)$ planes
C	Ga_0.8_In_0.2_N	Ga_0.8_In_0.2_N	Ga_0.8_In_0.2_N	(0001), $\left(1\bar{1}01\right)$, and $\left(10\bar{1}0\right)$ planes

## 3 Results and Discussion

### 3.1 Effect of Current Injection Positions in NW‐LEDs

To evaluate the current injection properties of NW‐LEDs with ITO electrodes, Devices p1, p2, and p3 with a mesa area of 340 × 340 μm^2^ were prepared from Sample p. Figure 2a presents an SEM image of the device structure after electrode fabrication, where the red region corresponds to NWs coated with ITO and the green region marks the mesa boundary. The ITO layer exhibits limited uniformity, suggesting the need for process optimization. All three devices were fabricated simultaneously and showed comparable surface morphologies. EL spectra of Devices p1, p2, and p3 under various injection currents are presented in Figure 2b. Despite identical fabrication processes, the three devices exhibit distinct spectral behaviors, indicating a strong dependence of emission characteristics on current distribution within the NWs. For Device p1, a dominant emission peak at around 708 nm arises primarily from In‐rich clusters and deep‐level defects, as carrier injection was confined to the (0001) polar plane due to thick ITO coverage on the top region. In Device p2, the emission may gradually shift from the (0001) plane to the $\left(1\bar{1}01\right)$ and $\left(10\bar{1}0\right)$ planes as the injection current increases. At 10 mA, a 759‐nm peak corresponds to the deep‐level defects or In‐rich clusters on (0001) polar plane region, whereas above 25 mA, two additional peaks at 686 and 746 nm appear from the $\left(1\bar{1}01\right)$ and $\left(0001\right)$ planes, respectively. At an injection current of 100 mA, two pronounced emissions around 560 and 674 nm dominate, with a weak residual peak at 752 nm, which may correspond to recombination on the $\left(10\bar{1}0\right)$, $\left(1\bar{1}01\right)$, and (0001) plane, respectively. Device p3 exhibits a distinct profile, showing a strong emission peak at 524 nm and a shoulder near 660 and 750 nm, corresponding to recombination on the $\left(10\bar{1}0\right)$, $\left(1\bar{1}01\right)$, and (0001) planes, respectively. The coexistence of multiple peaks indicates inhomogeneous current injection across different planes of the NWs, likely resulting from nonuniform ITO deposition.

**FIGURE 2:**
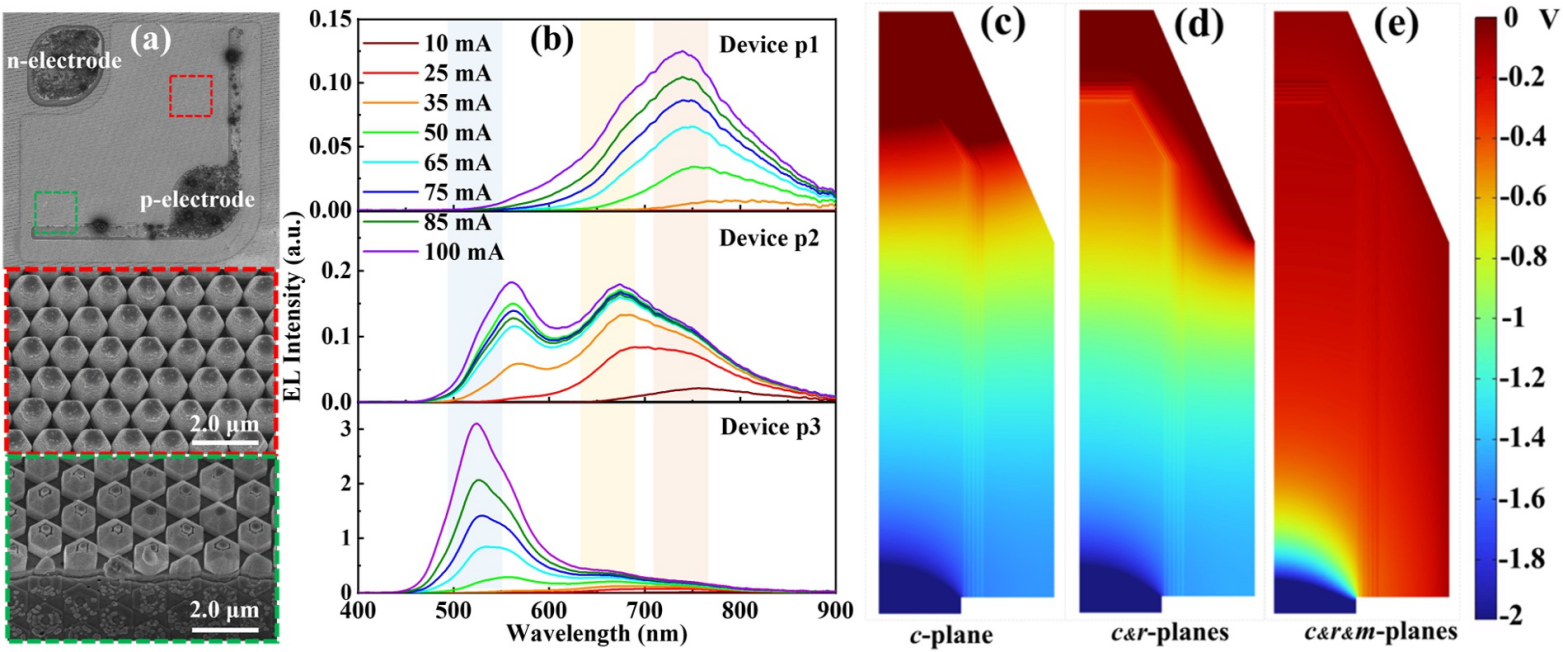
(a) Planar‐view SEM image of NWs in Sample p after electrode deposition. The red region within the mesa corresponds to NWs coated with ITO, whereas the green region indicates the mesa edge. (b) EL spectra of Devices p1, p2, and p3 acquired at injection currents of 10, 25, 35, 50, 65, 75, 85, and 100 mA. (c–e) Simulated potential distributions at a current density of 1000 A/cm^2^ for Samples A, B, and C, respectively.

To elucidate the current injection characteristics under different electrode configurations, NW‐LEDs were modeled using the finite element method. Figure 2c–e presents the simulated potential distributions under a current density of 1000 A/cm^2^ for three distinct p‐type contact configurations. When the electrode is deposited solely on the (0001) plane, current primarily diffuses axially from the top to the bottom of the NW, resulting in a concentrated injection path and nonuniform current distribution. This behavior is reinforced by the strong polarization fields along the (0001) plane, which restrict lateral carrier injection. When the electrode extends to both the (0001) and (1$\bar{1}$01) planes, part of the current is laterally injected through the (1$\bar{1}$01) planes, allowing transverse diffusion into the NW and partially improving spatial uniformity. However, on the (10$\bar{1}$0) plane, current still predominantly flows axially due to local potential gradients and higher contact resistance. Further extending the electrode to cover the (0001), (1$\bar{1}$01), and (10$\bar{1}$0) planes enables both axial and lateral injection from the sidewalls, enabling multidirectional carrier transport.

Figure 3a–c presents the spatial distribution of radiative recombination rates for the three p‐type contact configurations at 1000 A/cm^2^. The emission characteristics of core–shell NW‐LEDs strongly depend on electrode placement. When the p‐type contact is restricted to the (0001) plane, as shown in Figure 3a‐1,a‐2, radiative recombination is nearly suppressed on this plane, causing highly nonuniform emission and reduced overall device uniformity. Extending the electrode to include the (1$\bar{1}$01) and $\left(10\bar{1}0\right)$ planes results in relatively uniform recombination across all quantum well layers, as shown in Figure 3b‐1,b‐2,c‐1,c‐2. This improvement is attributed to enhanced lateral current spreading along the NW sidewalls, which mitigates local current crowding and potentially suppresses nonradiative recombination in the (0001) plane region. Figure 3d–f illustrates the corresponding electric field distributions under the same injection conditions. For the single‐plane (0001) contact, the polarization‐induced field aligns with the externally applied bias, intensifying the total field and suppressing radiative recombination on the (0001) plane, while introducing strong spatial inhomogeneity. When the electrode covers both the (0001) and $\left(1\bar{1}01\right)$ planes, the field distribution becomes more uniform, and its intensity decreases, facilitating more isotropic carrier injection. Further extending the contact to all three planes—(0001), $\left(1\bar{1}01\right)$, and $\left(10\bar{1}0\right)$—homogenizes the electric field, enabling multidirectional current spreading and uniform radiative recombination throughout the NW. These results demonstrate the mechanisms discussed in Figure 2, confirming that uniform electrodes can enhance lateral current distribution and mitigate polarization‐induced emission suppression. Therefore, multidirectional injection promotes electrical uniformity and uniform radiative recombination throughout the NWs.

**FIGURE 3:**
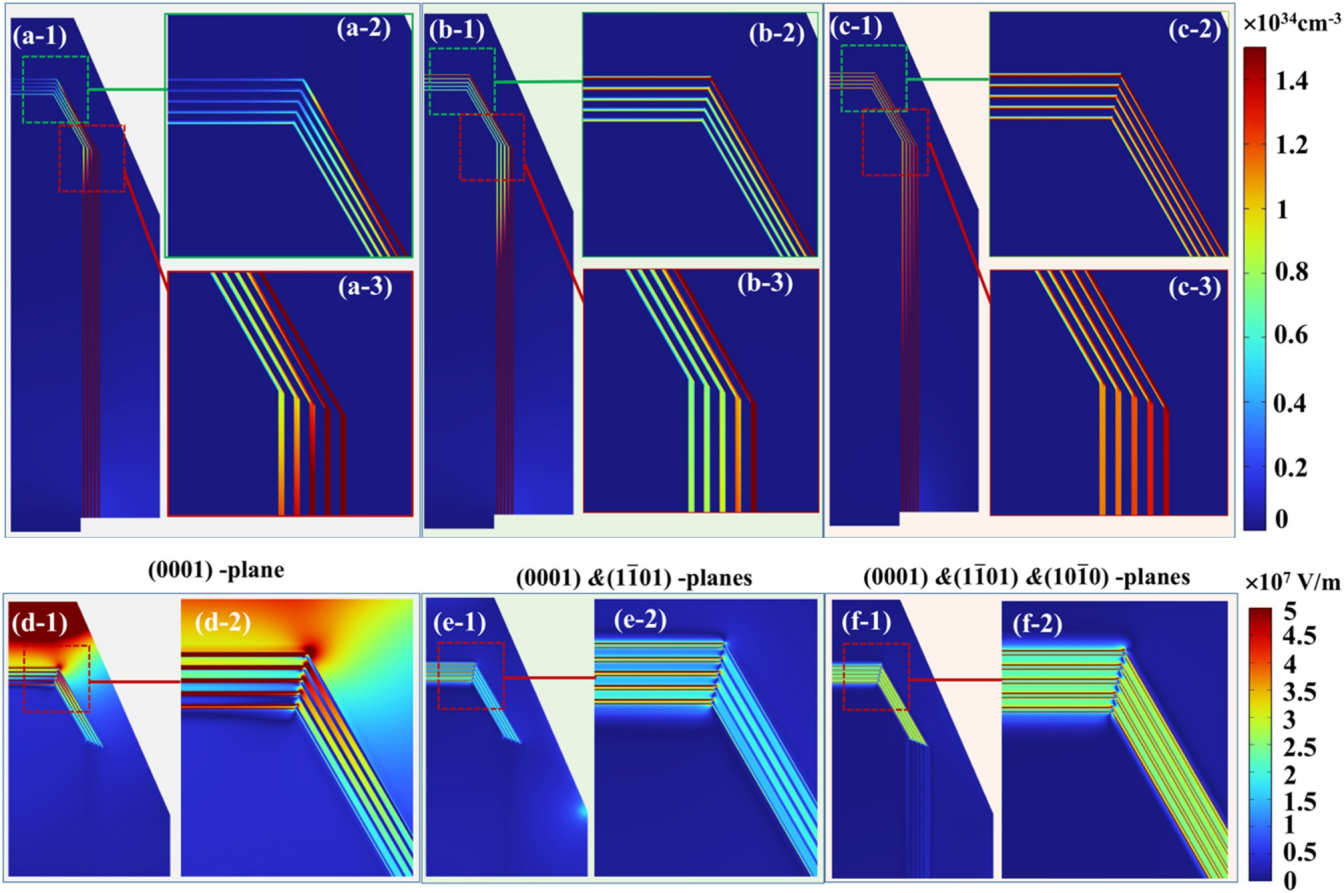
(a–c) Radiative recombination rate distributions of NW‐LEDs at 1000 A/cm^2^: (a–c‐1) overall mappings of Samples A–C; corresponding junctions (a–c‐2) between the (0001) and (1$\bar{1}$01) planes and (a–c‐3) between the $\left(1\bar{1}01\right)$ and $\left(10\bar{1}0\right)$ planes. (d–f) Electric field distributions of NW‐LEDs at 1000 A/cm^2^: (d–f‐1) overall mappings of Samples A–C, and (d–f‐2) corresponding junction between the (0001) and (1$\bar{1}$01) planes.

### 3.2 Comparison of ITO and AZO on the NW‐LED Structures

To improve sidewall electrode uniformity and mitigate the previously observed nonuniform current distribution, ALD‐deposited AZO was introduced. Figure 4 presents schematic diagrams and corresponding SEM characterization verifying the fabrication process of NW‐LEDs with ALD‐AZO. Figure 4a,b shows the device structure after AZO deposition on the NWs via ALD. In Figure 4b, the red‐highlighted boundary marks the interface between the mesa region and the etched surface, confirming the conformal AZO coverage. The blue region, where the AZO was deposited on the resist, exhibits a distinct geometric pattern in the SEM image, suggesting possible resist modification during ALD. Because the ALD process temperature was 200°C, conventional resists may have undergone thermal alteration. Figure 4c,d depicts the schematic and SEM images after resist removal. The extended stripping duration of the resist here was associated with the dense and conformal AZO film, which limited the effective contact between the resist and the developer solution. Additionally, the high ALD temperature likely altered the resist properties, resulting in incomplete removal. The red‐framed region in Figure 4d shows residual AZO along the mesa edge and partial resist residues at the bottom of the NWs. Meanwhile, in the blue region corresponding to the etched surface, the resist removal is evident with visible NW traces. These observations indicate that the patterning of AZO using resist masks in ALD‐based NW‐LED fabrication introduces process complexities due to the high deposition temperature and the inherently conformal nature of ALD coatings.

**FIGURE 4:**
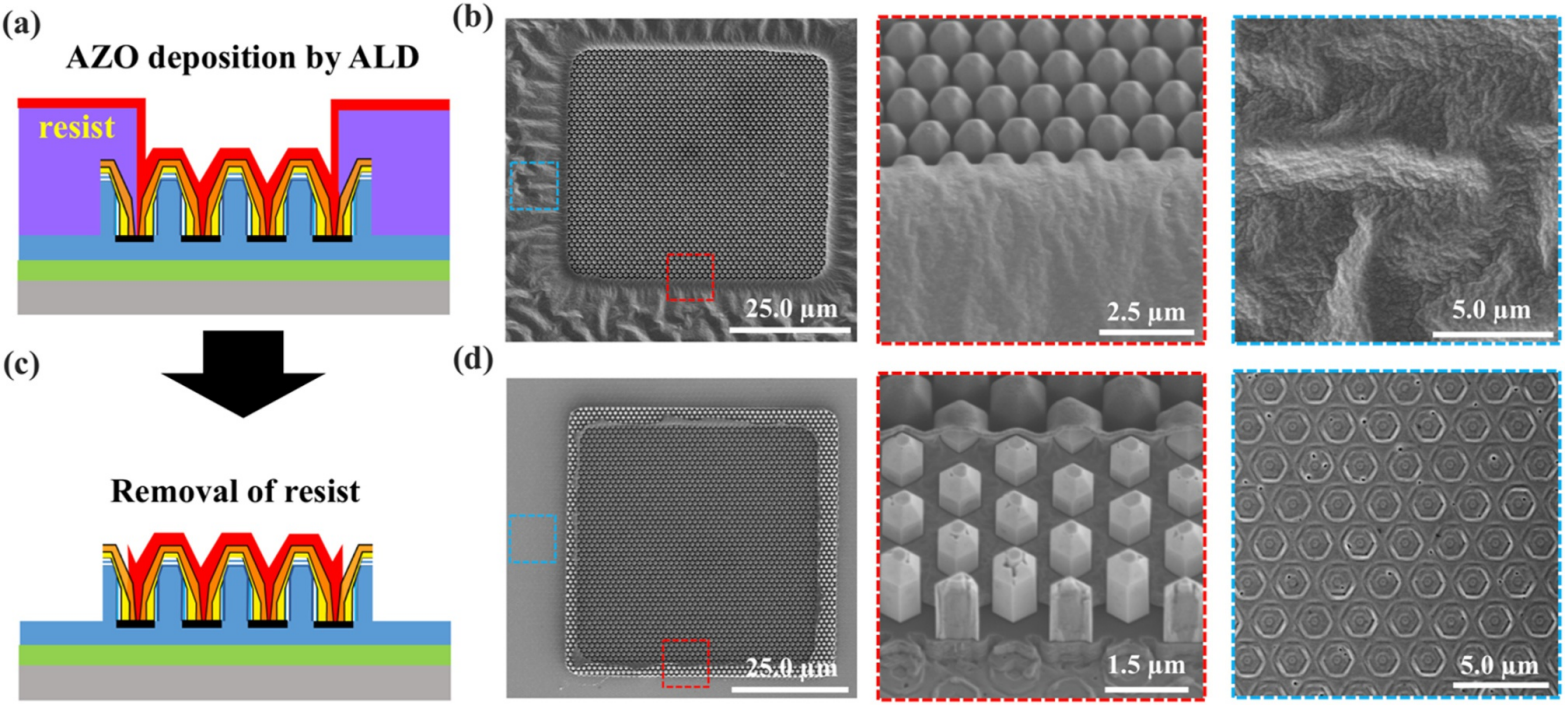
(a) Schematic diagram of AZO deposition by ALD in Sample a and (b) the corresponding SEM image. The red square area shows the edge of the mesa region, and the blue area at the etched surface corresponds to the n‐electrode. (c) Schematic diagram after removal of AZO. (d) SEM image of the device and the enlarged view of the mesa edge region (red square) and the etched surface corresponding to the n‐electrode (blue square).

To compare the transparent electrodes deposited on NWs by ALD and sputtering, Figure 5a presents planar SEM images of Samples a and b with AZO and ITO, respectively, whereas Figure 5b shows their corresponding cross‐sectional views. The cross‐sectional SEM images indicate that the NW morphology remains well preserved after ITO sputtering, with no observable structural degradation or deformation. The NW diameter in Sample a slightly increases after AZO deposition compared to Sample b with ITO. Cross‐sectional SEM analysis reveals uniform AZO layer thicknesses of 111, 118, and 120 nm on the (0001), ($1\bar{1}01$), and ($10\bar{1}0$) planes, respectively. In contrast, the ITO layer in Sample b is significantly thicker on the (0001) and ($1\bar{1}01$) planes than on the ($10\bar{1}0$) plane. This is inevitable because the configuration of the sputtering equipment emits the target materials from the top, leading to uneven deposition. Overall, for NW samples, ALD demonstrates superior consistency and thickness uniformity in AZO deposition compared to the sputtering method used for ITO. The normalized EL and I–V–L characteristics of Samples a and b are shown in Figure 5c,d. Sample a exhibits emission peaks at 465 and 485 nm with a subpeak at 608 nm, whereas Sample b shows a dominant peak at 526 nm and a subpeak at 633 nm, consistent with Section 3.1. The emission wavelength variation is attributed to the NW height dependence, where shorter wavelengths originate from the lower regions of the NWs [45]. Accordingly, the bottom emission dominates in Sample a, producing a blue luminescence, whereas Sample b exhibits green luminescence. From the I–V–L results in Figure 5d, the operating voltage at 3 mA was 3.6 V for Sample b and 16.7 V for Sample a. Despite the high operating voltage, the light output of Sample a was 5.4 times higher, suggesting enhanced carrier injection into the lower NW regions. These findings confirm that ALD‐grown AZO electrodes improve emission intensity through more uniform sidewall coverage. However, further optimization of the device structure and AZO/p‐GaN contact resistance is required to reduce the operating voltage in NW‐LEDs.

**FIGURE 5:**
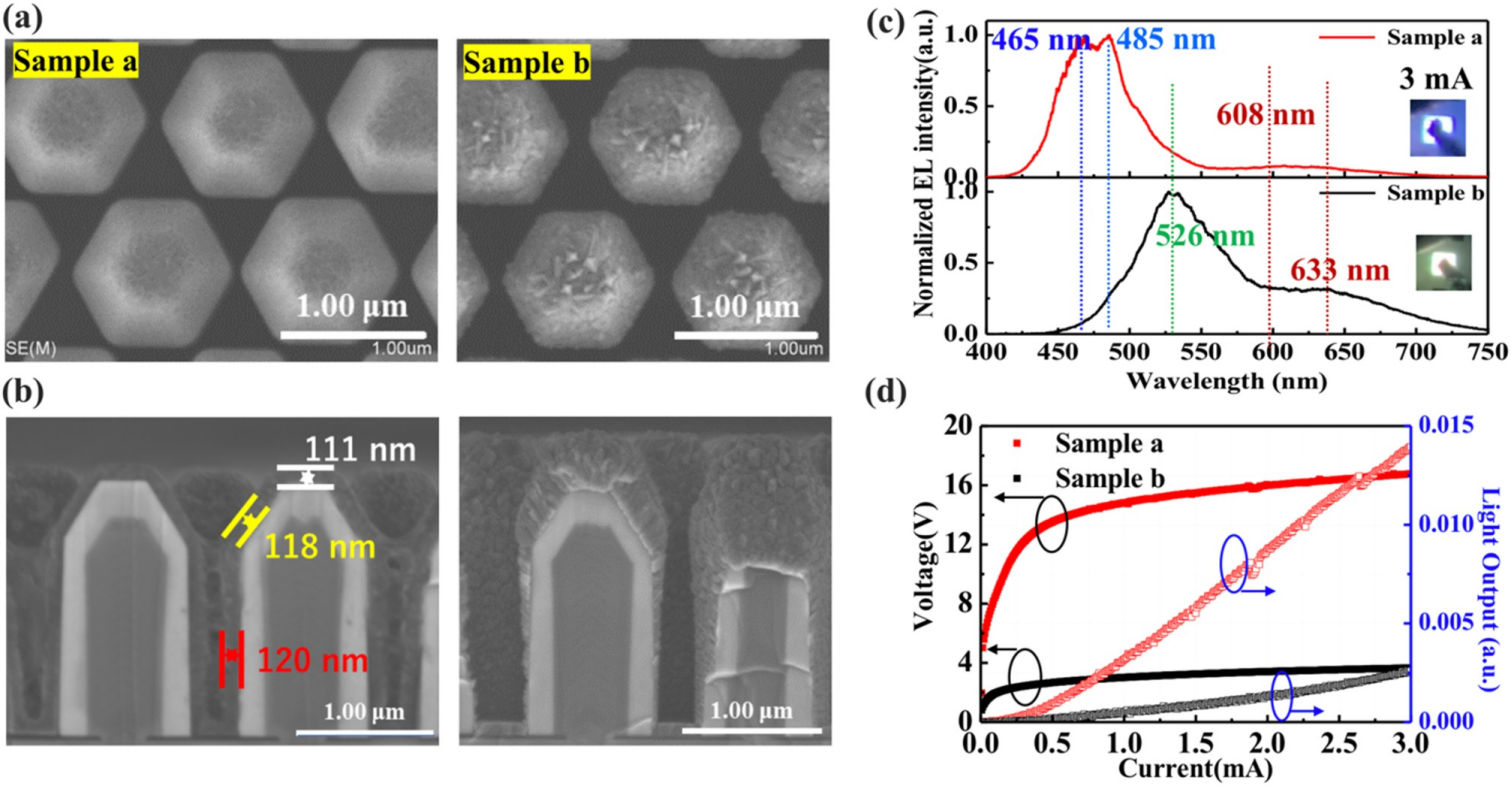
(a) Planar‐ and (b) cross‐sectional‐view SEM images of Samples a and b. (c) EL spectra at an injection current of 3 mA. Insets show the emission photos captured under a 3‐mA injection current. (d) I–V–L characteristics of Samples a and b.

### 3.3 Modified Fabrication Process of NW‐LEDs for Reducing Operating Voltage

For Samples a and b, AZO patterning was initially carried out using a photoresist lift‐off process after the ALD process. However, due to the conformal deposition behavior of ALD and the elevated deposition temperature of 200°C, the resist stripping process became difficult, and resist‐related residues near the mesa boundary may also increase the operating voltages. To further validate the NW‐LED fabrication process and minimize the influence of boundary NWs and process‐induced edge effects, an optimized device structure employing dry etching for AZO patterning was developed, as illustrated in Figure 6a. For Sample c, AZO was deposited on the entire surface by ALD following mesa formation. Subsequently, the AZO outside the mesa region was selectively removed using a resist mask and BCl_3_‐based ICP dry etching. To further suppress unintended current injection and reduce the influence of edge‐related effects, such as dry‐etching damage and nonuniform carrier injection near the mesa boundary, a SiO_2_ isolation layer was introduced along the mesa edge prior to AZO deposition. After AZO patterning, the n‐electrode was deposited on the etched surface, whereas the p‐electrode was formed on the NWs within the mesa region. This optimized fabrication process enables a more reliable evaluation of the intrinsic current spreading and carrier injection characteristics of the ALD‐AZO transparent electrode. Figure 6b shows an SEM image of the completed device. The red region indicates the NWs coated with ALD‐AZO, the green region marks the AZO etching boundary, and the blue area denotes the mesa edge. The SEM image in the red region confirms that the AZO within the mesa remained intact during etching, owing to the protection provided by the resist mask. The green region shows successful AZO patterning via dry etching, whereas the blue region confirms that no unwanted AZO‐to‐n‐GaN contact occurred, preventing potential leakage paths. These results demonstrate that AZO can be effectively patterned by dry etching while maintaining electrical isolation and minimizing leakage risks in NW‐LED fabrication.

**FIGURE 6:**
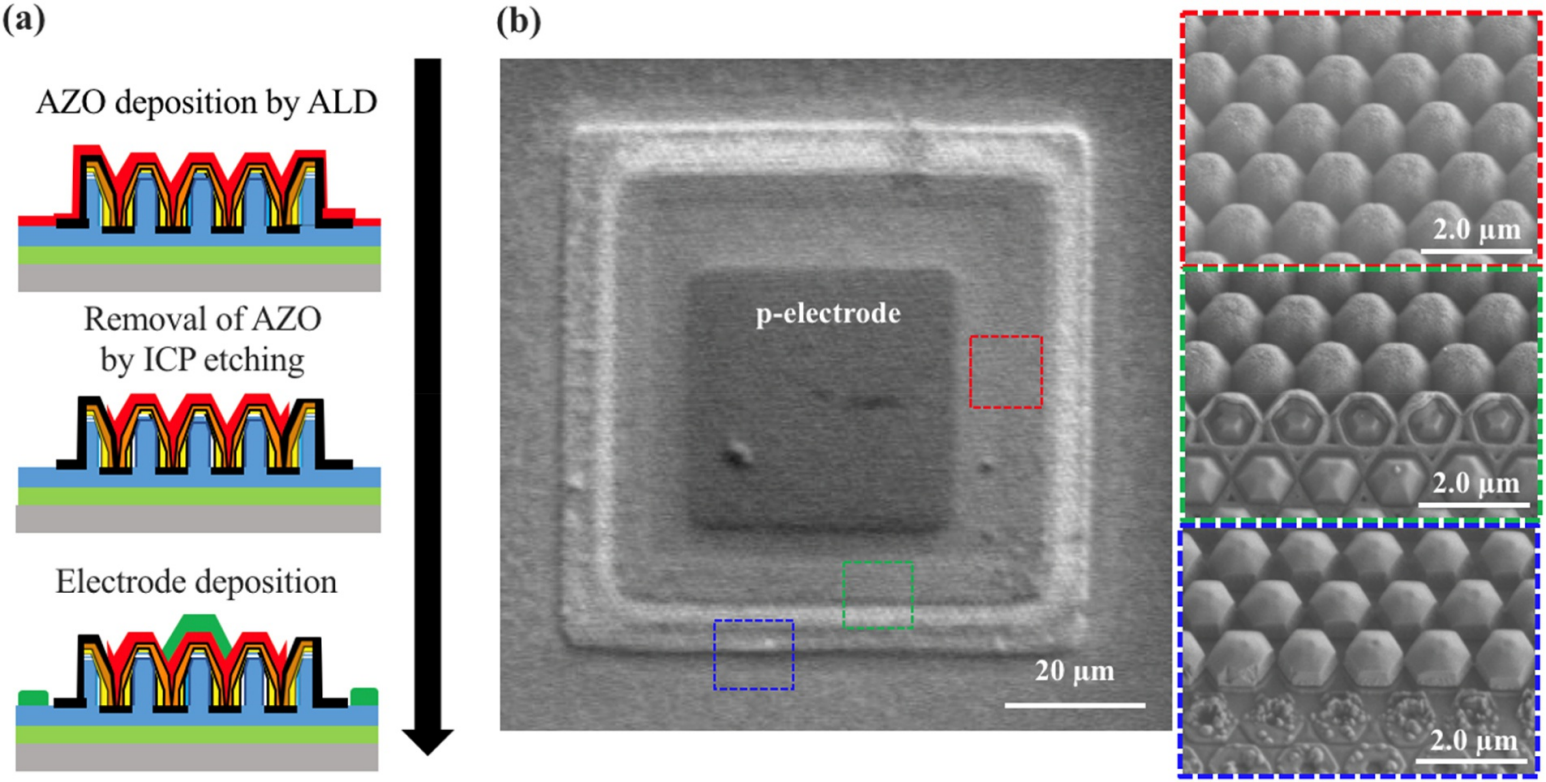
(a) Process flow of transparent and pad electrode formation. (b) SEM images of Sample c with electrode deposition, showing the general view. The red area is the NWs covered with ALD‐AZO, the green area is the etched boundary of AZO, and the blue area is the edge of the mesa area.

Figure 7a depicts cross‐sectional SEM images of NWs in Samples c and d after AZO and ITO deposition, respectively. In Sample c, the AZO thicknesses were measured as 124 nm on the (0001) plane, 140 nm on the ($1\bar{1}01$) plane, and 149 nm on the ($10\bar{1}0$) plane. For Sample d, the ITO layer measured 183 nm on the (0001) plane and 125 nm on the ($1\bar{1}01$) plane, whereas its thickness on the ($10\bar{1}0$) plane was too thin to quantify, consistent with the results in Section 3.2. Notably, the AZO film deposited by ALD also showed greater coverage on the SiO_2_ mask between NWs, suggesting improved current spreading across the chip in Sample c. Figure 7b shows the EL spectra and emission photographs of Samples c and d. Figure 7c replots the emission peak and FWHM as a function of the injection current (1–5 mA). At 5 mA, Sample c exhibited a peak wavelength of 492 nm with a FWHM of 45 nm (blue emission), whereas Sample d emitted at 580 nm with a FWHM of 83 nm (orange emission). Previous cathodoluminescence studies have shown that the lower regions of the NWs exhibit shorter emission wavelengths owing to facet‐dependent In incorporation [21]. Therefore, the shorter emission wavelength and narrower FWHM observed in Sample c suggest enhanced emission from the lower and sidewall regions of the NWs. This behavior is consistent with the superior conformal coverage of the ALD‐AZO electrode, which promotes more uniform current injection throughout the entire NW structure and facilitates carrier transport to regions that are less effectively contacted by sputtered ITO. With increasing injection current, Sample d displayed a gradual blueshift in its emission peak, whereas Sample c remained spectrally stable. This behavior aligns with previous reports that higher current densities extend the emission region from the NW apex toward the sidewalls [46, 47]. The luminescence photographs also reveal that at 2 mA, Sample d exhibited uniform emission across the mesa, whereas Sample c showed localized bright spots at 1 mA. This indicates that despite the conformal AZO coverage achieved by ALD, current crowding at specific regions led to spot‐like emission. It is believed that the high current density at localized points enhanced emission at the bottom of the NWs. Figure 7d shows the I–V–L characteristics of Samples c and d. At an injection current of 5 mA, the operating voltages of Samples c and d were 6.7 and 2.9 V, respectively, whereas the light output of Sample c was 2.8 times higher than that of Sample d. These results are consistent with those discussed in Section 3.2. Compared with Samples a and b (50 × 50 μm^2^), the larger device area for Samples c and d (100 × 100 μm^2^) can slightly reduce the operating voltage because of the lower current density. However, as demonstrated in our previous work, mesa‐size effects mainly influence the carrier injection pathway and emission behavior, whereas their effect on operating voltage is relatively limited [20]. More importantly, Samples c and d were fabricated with identical mesa sizes and device geometries, differing only in the transparent electrode material and deposition method. Under these conditions, the ALD‐AZO device still exhibited significantly enhanced light output and improved emission uniformity compared with the sputtered‐ITO device. These results further demonstrate that the improved optical performance primarily originated from the superior conformal coverage and enhanced current spreading capability of the ALD‐AZO electrode.

**FIGURE 7:**
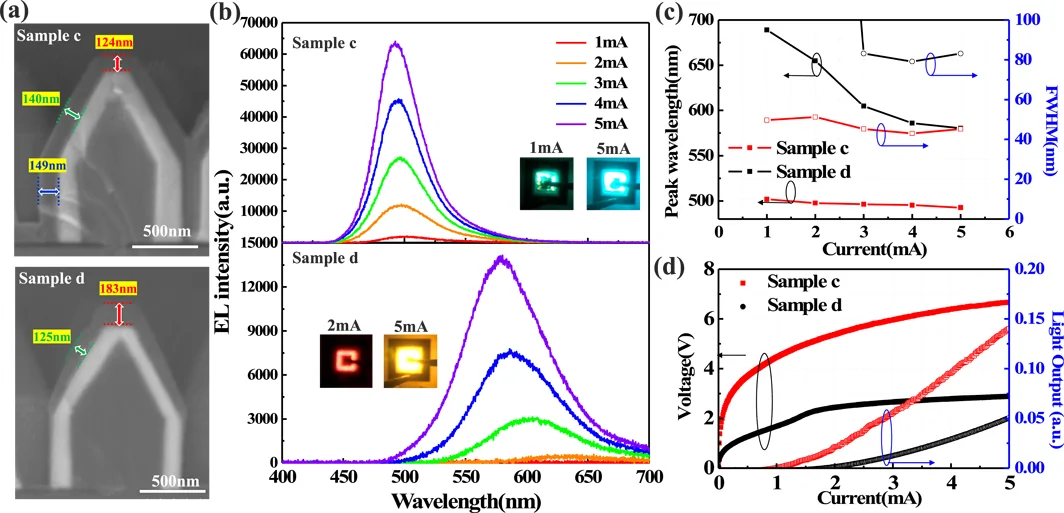
(a) Cross‐sectional SEM images of Samples c and d. (b) EL spectra acquired at injection currents of 1, 2, 3, 4, and 5 mA. Insets show the captured emission photographs. (c) Emission peak and full width at half maximum (FWHM) as a function of injection current varying from 1 to 5 mA. (d) I–V–L characteristics of Samples c and d.

### 3.4 Effect of the Thermal Annealing of AZO on the Emission Properties of NW‐LEDs

To evaluate the electrical properties of the ALD‐AZO/p‐GaN contact, AZO films were deposited onto planar LED structures and characterized using circular TLM patterns. Postdeposition thermal annealing was carried out to investigate its influence on the electrical characteristics. As illustrated in Figure 8a, four samples (A, B, C, and D) were prepared for analysis. Sample A served as the as‐deposited AZO reference without annealing. Samples B and C with 150 nm‐thick AZO films were annealed at 600°C and 650°C, respectively, for 4 min under a N_2_ atmosphere using RTA. Sample D, incorporating a 150‐nm‐thick ITO layer, was annealed at 600°C for 4 min under identical conditions for comparison. Figure 8b illustrates the planar view of the circular TLM structure, whereas Figure 8c shows the corresponding cross‐sectional schematic. The AZO and ITO electrodes were deposited on p‐GaN with a spacing of 5 μm between adjacent electrodes. Figure 8d presents the measured V–I characteristics. Among all samples, the ITO‐based Sample D exhibited the lowest resistance, whereas the as‐deposited AZO sample showed significantly higher resistance. After thermal annealing, the resistance of the AZO samples decreased markedly with increasing annealing temperature, indicating improved electrical conduction and enhanced contact formation at the AZO and p‐GaN interface. These results indicate that the relatively high operating voltages observed in the ALD‐AZO‐based NW‐LEDs discussed in Sections 3.2 and 3.3 mainly originated from the high resistance of the as‐deposited AZO layer and the nonoptimized AZO/p‐GaN contact.

**FIGURE 8:**
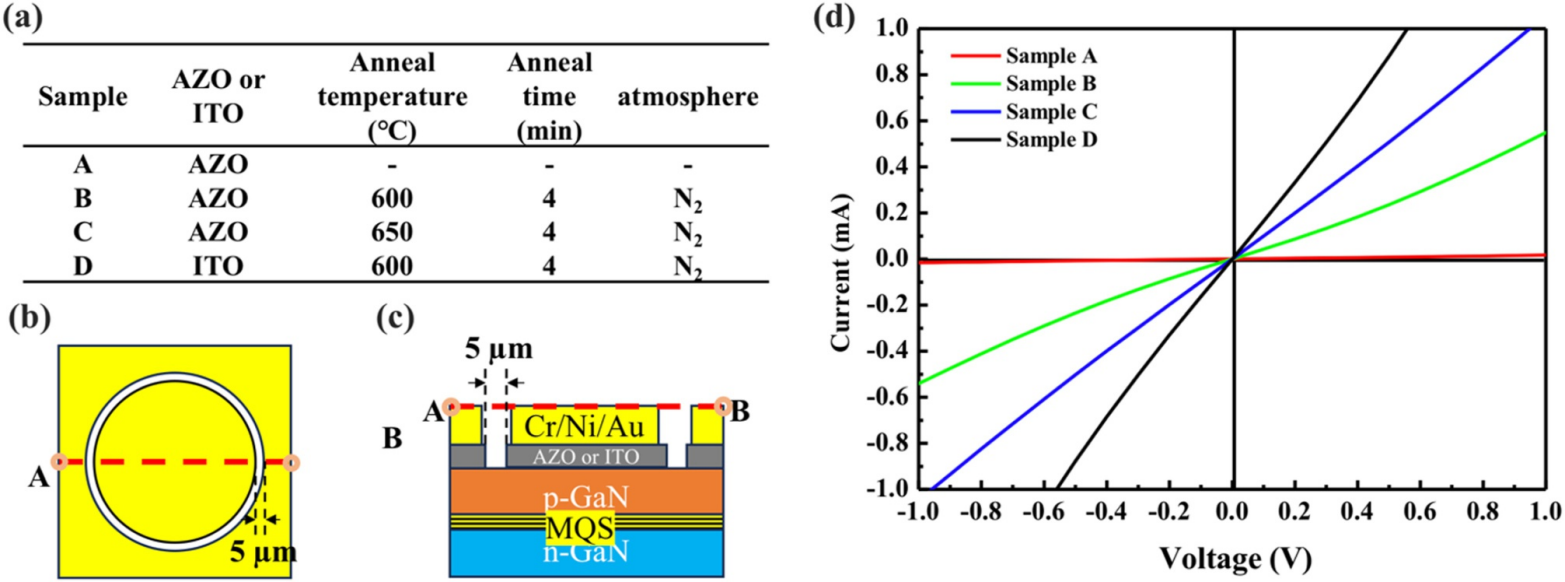
(a) Fabricated planar LED samples with different annealing conditions of AZO. (b) Planar and (c) cross‐sectional schematic structures of the circular TLM structure. (d) Measured V–I characteristics of Samples A, B, C, and D.

EL spectra and I–V–L characteristics were analyzed to assess the device performance of samples with annealed AZO. Sample e with AZO annealed at 650°C for 4 min in a N_2_ atmosphere and Sample f using ITO were comparatively investigated. Figure 9a,b shows the EL spectra and corresponding emission photographs captured at 3, 6, 15, 24, and 30 mA, respectively. The emission intensity in Sample e was clearly improved at injection currents above 3 mA. At 30 mA, Sample e exhibited a dominant emission peak at 492 nm, whereas Sample f showed a similar peak wavelength around 496 nm. Because the shape of the NWs was optimized by inserting 40 pairs of SLs and extending the growth time of barriers, the conformality of sputtered ITO on NWs was also improved [21, 36]. Therefore, carrier injection into the sidewall regions was enhanced even in Sample f. Figure 9c presents the corresponding peak wavelength and FWHM as a function of the injection current. At 30 mA, the FWHM values of Samples e and f were 39.4 and 46.4 nm, respectively. The narrower emission linewidth observed in Sample e indicates more dominant emission from the lower regions of the NWs at high injection currents. Such behavior is attributable to the superior conformal coverage of the ALD‐AZO electrode, which enables more efficient current injection into the bottom and sidewall regions of the three‐dimensional NW structure. Figure 9d shows the corresponding I–V–L characteristics. Similar to the low‐current injection region, Sample e maintained a lower operating voltage than Sample f over the entire injection range, confirming the effectiveness of thermal annealing in improving the electrical properties of the AZO electrode. At low injection currents below 7.5 mA, Sample f exhibited slightly higher light output than Sample e. However, as the injection current increased further, the light output of Sample e exceeded that of Sample f. At 30 mA, the light output of Sample e was approximately 1.2 times higher than that of Sample f. This current‐dependent behavior suggests that, at low current densities, carrier injection was mainly concentrated near the top region of the NWs [48], where the influence of sidewall electrode coverage was relatively limited. As the injection current increased, carriers progressively spread toward the lower and sidewall regions of the NWs. Therefore, the highly conformal ALD‐AZO electrode provides more uniform current injection throughout the entire NW structure, resulting in enhanced radiative recombination and improved light output.

**FIGURE 9:**
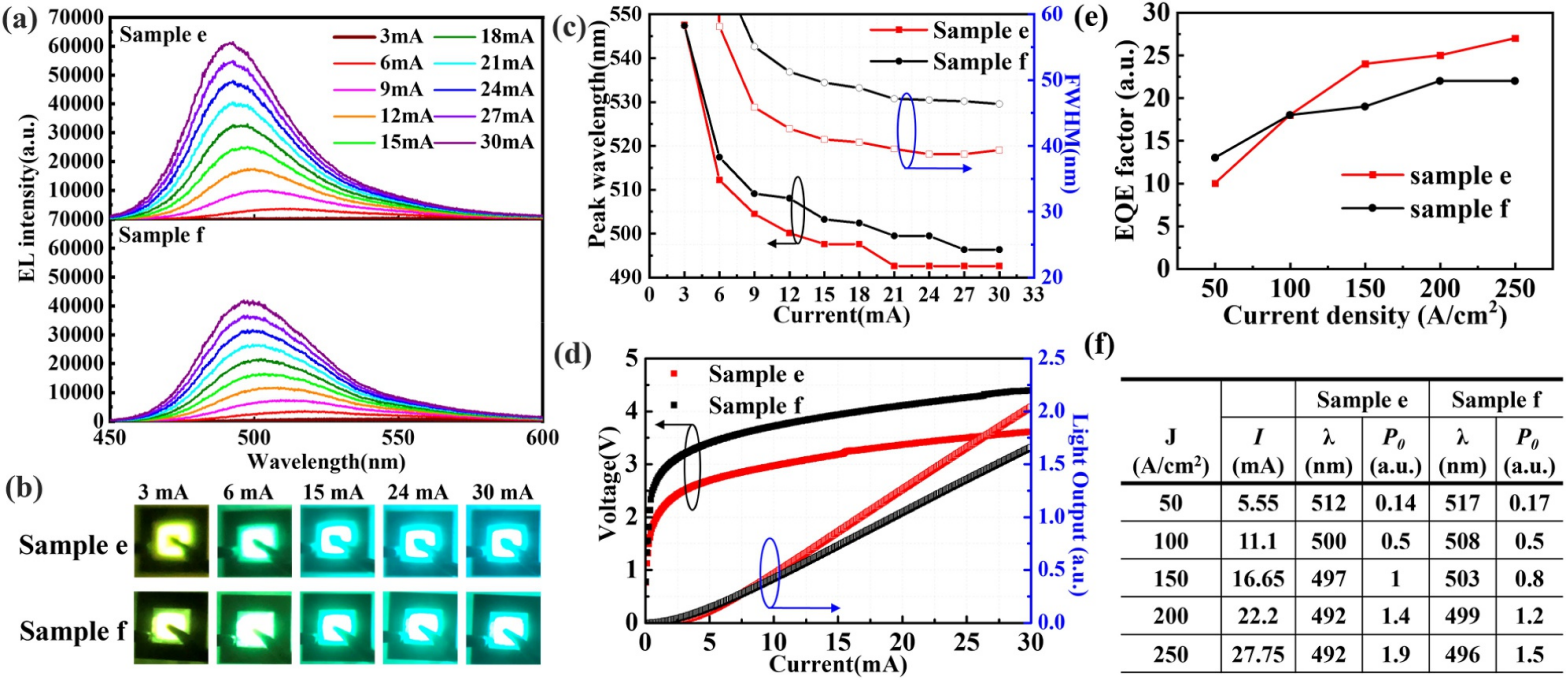
(a) EL spectra of Samples e and f. (b) Emission photos under injection currents of 3, 6, 15, 24, and 30 mA. (c) Emission peak and FWHM as a function of injection current varying from 3 to 30 mA. (d) I–V–L characteristics of Samples e and f. (e) The parameters of current (*I*), wavelength (*λ*), and photons emitted per second (*P*_0_) were derived from the measurement results. (f) Estimated EQE factor as a function of the emission area of the NW‐LEDs.

External quantum efficiency (EQE) is one of the most important parameters for evaluating LED device performance [4, 49]. Although the light output of the NW‐LEDs was detected from the backside of the substrate, an approximate EQE factor was derived to evaluate and compare the emission characteristics of the devices [50]:

$$\text{EQE}\;\text{factor}\left[a.u.\right]=\frac{P_{0}}{I}\times \frac{\lambda e}{\text{hc}},$$

where *h* is Planck's constant, *e* is the elementary charge, *c* is the speed of light, *P*_0_ is the optical output intensity (a.u.) measured by the mini‐spectrometer, *I* is the injection current, and *λ* is the peak emission wavelength. The EQE factor was calculated at current densities of 50, 100, 150, 200, and 250 A/cm^2^. Figure 9e,f presents the extracted experimental results from I–V–L characteristics and calculated EQE factors. At injection current densities below 100 A/cm^2^, the emission mainly originates from the top region of the NWs. As the current density increased, carrier injection gradually extended toward the semipolar and nonpolar facets, resulting in enhanced emission from the sidewall regions. Owing to the superior conformal coverage and enhanced current spreading provided by the ALD‐AZO electrode, the AZO‐based device exhibited a higher EQE factor than the ITO‐based device, with an enhancement of approximately 1.2 times. These results indicate that the conformal deposition of AZO by ALD promotes more uniform current injection throughout the entire NW structure, particularly in the lower and sidewall regions that are difficult to effectively contact using sputtered ITO.

## 4 Conclusion

In summary, we systematically investigated the fabrication process, transparent electrode design, and optoelectronic performance of GaInN/GaN NW‐LEDs employing two different transparent electrodes and deposition methods. Sputtered ITO exhibited nonuniform sidewall coverage, leading to spatial variations in emission spectra. Finite element simulations confirmed that improved electrode coverage effectively improved lateral current spreading, promoting uniform carrier transport along NW sidewalls and alleviating emission nonuniformities caused by polarization‐induced spatial fluctuations. In contrast, ALD‐grown AZO exhibited excellent conformality and uniform thickness across NW planes. Two fabrication processes, namely, resist lift‐off and selective AZO dry etching after mesa formation, were successfully demonstrated for electrode patterning. Both patterning methods demonstrated selective AZO deposition on the mesa region, though the resist mask was affected by the ALD operating temperature. Device characterization showed that ALD‐AZO electrodes significantly enhanced current injection into the lower regions of the NWs, resulting in improved emission uniformity, higher light output, and increased EQE factor compared with sputtered ITO devices. Although the as‐deposited AZO devices exhibited relatively high operating voltages, circular TLM measurements and postdeposition annealing studies confirmed that the AZO/p‐GaN contact resistance can be substantially reduced through thermal treatment. After optimization, the annealed ALD‐AZO devices exhibited lower operating voltages than the corresponding ITO devices while maintaining superior optical performance. At an injection current of 30 mA, the ALD‐AZO NW‐LEDs achieved a 1.2‐fold enhancement in light output compared with their ITO counterparts. These results demonstrate that conformal ALD‐AZO electrodes effectively improve current‐spreading uniformity, carrier injection, and emission efficiency in three‐dimensional NW‐LEDs.

## Author Contributions

**Soma Inaba:** methodology, data curation, conceptualization, writing – original draft, investigation, formal analysis. **Mengtong Wang:** validation, data curation, formal analysis, writing – original draft, software. **Weifang Lu:** conceptualization, investigation, methodology, funding acquisition, writing – review and editing, validation, supervision, formal analysis, data curation. **Kazuma Ito:** investigation, methodology, visualization, data curation. **Sena Miura:** investigation, validation, methodology. **Yiyu Ou:** investigation, methodology, formal analysis. **Dong–Pyo Han:** investigation, formal analysis, validation. **Kai Huang:** funding acquisition, formal analysis, data curation. **Motoaki Iwaya:** funding acquisition, methodology, conceptualization, project administration. **Tetsuya Takeuchi:** methodology, visualization, funding acquisition, conceptualization, project administration. **Rong Zhang:** conceptualization, validation, project administration, formal analysis. **Satoshi Kamiyama:** conceptualization, validation, funding acquisition, writing – review and editing, supervision, resources, project administration, formal analysis.

## Funding

The authors from Xiamen University, China, would like to acknowledge the Advanced Materials‐National Science and Technology Major Project (Grant 2025ZD0616201) and the National Natural Science Foundation of China (Grant 62404184). This work was financially supported by MEXT “Program for research and development of next‐generation semiconductor to realize energy‐saving society” (Grant JPJ005357), MEXT “Private University Research Branding Project,” JSPS KAKENHI for Scientific Research A (Grant 15H02019), JSPS KAKENHI for Scientific Research A (Grant 17H01055), JSPS KAKENHI for Innovative Areas (Grant 16H06416), and Japan Science and Technology CREST (Grant 16815710). This work was also partially supported by the Global Joint Research Program funded by Pukyong National University (Grant 202505830001).

## Data Availability

The data that support the findings of this study are available upon request from the corresponding author. The data are not publicly available due to privacy or ethical restrictions.
